# Chondral defects of the glenohumeral joint: Long-term outcome after microfracturing of the shoulder

**DOI:** 10.1007/s11678-017-0415-3

**Published:** 2017-06-29

**Authors:** Sophia M. Hünnebeck, Petra Magosch, Peter Habermeyer, Markus Loew, Sven Lichtenberg

**Affiliations:** 1 Abteilung für Obere Extremität, Hand- und Mikrochirurgie, Immanuel Krankenhaus Berlin, Königstraße 63, 14109 Berlin, Germany; 2 German Joint Center Heidelberg, ATOS Clinic Heidelberg, Bismarckstraße 9–15, 69115 Heidelberg, Germany

**Keywords:** Arthroscopy, Osteoarthritis, Omarthritis, Pain, Arthroplasty, Arthroskopie, Arthritis, Omarthrose, Schmerz, Arthroplastik

## Abstract

**Introduction:**

An increasing number of young patients are diagnosed with chondral lesions. Minimally invasive surgical techniques are important in order to delay progression of the early stages of osteoarthritis and the need for total joint replacement.

**Materials and methods:**

Patients (*n* = 32) who had received microfracturing of the shoulder were retrospectively enrolled, of whom 5 had received shoulder replacements after a mean time of 47 months. Of these patients, 23 completed the Disabilities of the Arm, Shoulder and Hand (DASH) and Constant–Murley Scores in addition to an additional subjective questionnaire. Patients were then clinically examined and received x‑ray analysis of the operated shoulder. Data from an additional 4 patients were acquired by telephone interview.

**Results:**

Mean follow-up was 105 months. Of the included patients, 13/27 patients reported no pain, 12/27 patients moderate pain. Of these 12, 6/27 reported pain only at night and 3/27 only during rest. Concerning the outcome of surgery, 19/27 patients were “satisfied” or “very satisfied”. There was a statistically significant increase in internal rotation, but no further differences in the range of motion pre- and postoperatively. Patients without any signs of osteoarthritis before surgery showed statistically significantly better outcomes. There was a statistically significant increase in radiological signs of osteoarthrosis in pre- versus postoperative patients. Patients with bipolar lesions showed statistically significantly poorer Subjective Shoulder Value (SSV) results.

**Conclusion:**

Even though microfracturing does not prevent radiographic progression, microfracture of the glenohumeral joint might be worth considering as part of a treatment regimen for younger patients who may not yet be treated with arthroplasty.

## Zusammenfassung

**Hintergrund:**

Zunehmend werden bei jungen Patienten Knorpelläsionen diagnostiziert. Deren Therapie ist eine Herausforderung für den Behandler. Minimalinvasive Op.-Methoden sind wichtig, um eine Progression zur Omarthrose zu verzögern und einen frühen Gelenkersatz zu vermeiden.

**Material und Methoden:**

Retrospektiv wurden 32 Patienten, bei denen eine Mikrofrakturierung erfolgt war, in die Studie eingeschlossen. Nach im Mittel 47 Monaten hatten 5 davon einen Gelenkersatz erhalten. Mittels DASH und Constant-Murley-Score sowie subjektiver Fragebögen, klinischer Untersuchung und Röntgenbild wurden 23 Patienten nachuntersucht. Bei weiteren 4 Patienten wurden die Daten der Fragebögen telefonisch erfragt.

**Ergebnisse:**

Das durchschnittliche Follow-up lag bei 105 Monaten. Von 27 Patienten waren 13 schmerzfrei; 12 berichteten über moderate Schmerzen, davon 6 über Nacht- und 3 über Ruheschmerz. Mit dem Ergebnis sehr zufrieden oder zufrieden waren 19 Patienten. Die Innenrotation verbesserte sich statistisch signifikant im Vergleich zu vor der Op., darüber hinaus ergaben sich keine Änderungen des Bewegungsumfangs. Patienten, die zum Zeitpunkt der Op. keinerlei Zeichen der Arthrose im Röntgenbild aufwiesen, erreichten bessere Ergebnisse. Insgesamt war jedoch eine statistisch signifikante Zunahme der Omarthrose im Röntgenbild über die Zeit des Follow-up zu verzeichnen. Patienten mit intraoperativ bipolaren Läsionen wiesen im Subjective Shoulder Value (SSV) statistisch signifikant schlechtere Ergebnisse auf.

**Schlussfolgerung:**

Die Mikrofrakturierung des Schultergelenks bei lokalisierten Knorpelschäden verhindert eine radiologische Progression der Omarthrose nicht, dennoch ist die Op.-Technik als Teil einer multimodalen Therapie zu erwägen, um weitere, invasivere Therapieoptionen hinauszuzögern.

Finding the right treatment for a young patient with glenohumeral arthritis is still a challenge. It depends not only on the radiological findings but also on the explicit age of the patient, their occupation, activity level, duration of symptoms, comorbidities and concomitant shoulder pathology.

## Introduction

Glenohumeral arthritis is a condition that can be responsible for persistent shoulder pain and functional limitation compared to a healthy individual [7]. The occurrence of glenohumeral arthritis is less common than that of the knee or hip; however, the incidence of this pathology increases with the age of the population [5]. Accompanying the increasing rate of diagnostic tool development and subsequent detection of chondral lesions, the number of young patients diagnosed with early or even later stage osteoarthritis is growing [1].

Particularly in younger patients, diagnosing early glenohumeral arthritis is often a “diagnosis of exclusion” [1]. It has been reported that the incidence of symptomatic chondral lesions (Outerbridge grade II–IV) [16] in diagnostic arthroscopy can be up to 17% in middle-aged patients with full-thickness rotator cuff tears or in active overhead athletes [4, 11].

In case of failure of conservative therapy, surgical treatment must be considered. Various treatment options exist, such as local debridement, microfracturing, restorative techniques (autologous chondrocyte implantation, osteochondral autograft transplantation surgery), joint replacement or allografts [1, 19]. Since the long-term outcome—especially for the procedure of microfracturing of the shoulder—is still research in progress, it is important to have alternative treatment options to shoulder arthroplasty, particularly for patients younger than 60 years.

The aim of this study was to evaluate long-term outcomes following microfracture of the shoulder joint in patients with chondral lesions of the glenohumeral joint, by determining the prevalence of early stages of osteoarthritis during the follow-up period.

## Materials and methods

### Study design, patients

**Fig. 1:**
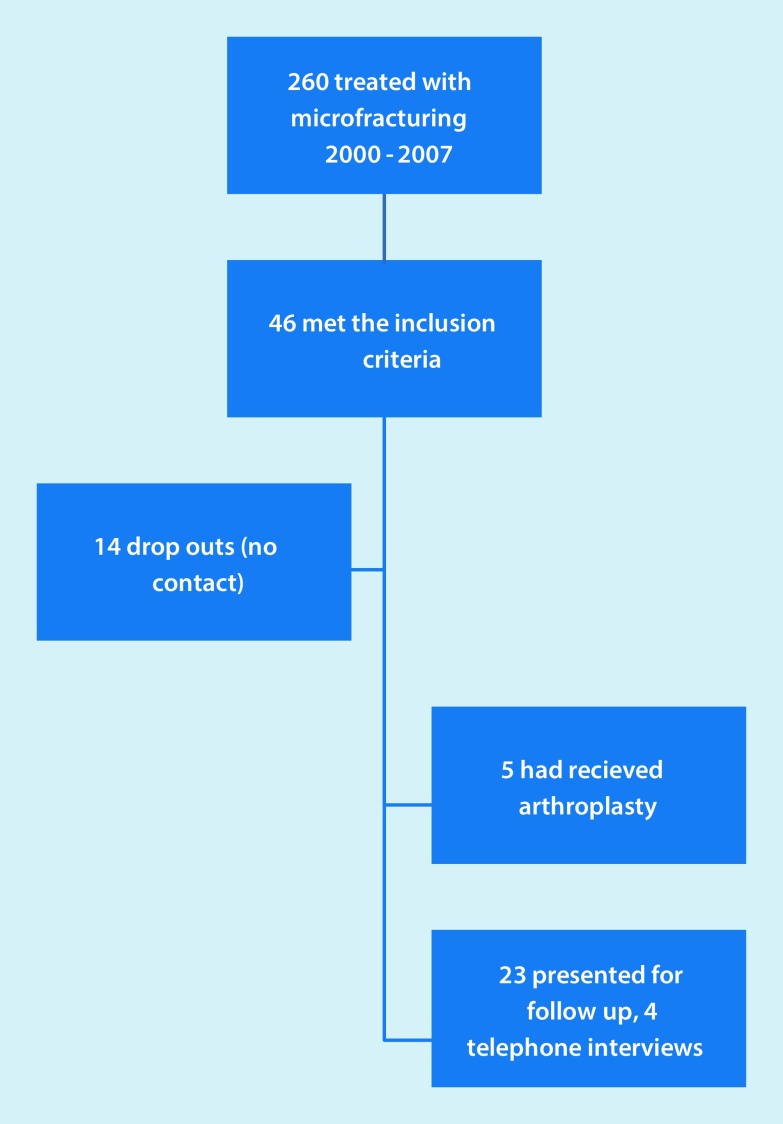
Flowchart describing the patient numbers at each stage

Inclusion criteria were: chondral defects of the humeral head, glenoid or both, which had been treated with arthroscopic microfracture. Exclusion criteria included history of glenohumeral instability, large rotator cuff tears, and fractures or systemic inflammatory diseases of the joint. See Fig. 1 for the patient numbers at each stage. Any kind of shoulder arthroplasty after microfracture of the shoulder was defined as an endpoint of the study; these patients were included in the results according to an intention-to-treat analysis. Of the included patients, 4 were not able to present personally and the questionnaires were verbally completed by telephone. The remaining 23 patients received a clinical examination and x‑ray and MRI of the shoulder. Constant-Murley Score (CMS), Disabilities of the Arm, Shoulder and Hand (DASH) Score and an additional questionnaire for subjective evaluation of the treated shoulder were obtained. All patients signed written consent to take part in this study. Included were 17 male (53%) and 15 female (47%) patients with an average age of 56 years (37–74). Regarding treatment, 32 shoulders were treated with microfracturing; 11 left shoulders (34%) and 21 right (66%) shoulders, of which the dominant shoulder was treated in 18 cases (56%). A minor trauma in the past was reported by 9 patients (28%) and 23 patients (72%) had no history of any trauma. Regarding previous surgery, 5 patients (16%) had been operated on once before the treatment and 1 patient had already been operated upon twice. Intraoperatively, 31 patients presented with a chondral lesion grade IV according to Outerbridge (subchondral bone) and 1 patient presented with grade III (fissuring of the cartilage) [9]. The initial indication for surgery was constant shoulder pain without satisfying effects of conservative treatment. In those cases without preoperative signs of osteoarthrosis in imaging, chondral lesions were suspected but first diagnosed during arthroscopy.

In total, 30 humeral lesions and 14 glenoid lesions were treated with microfracture. Of the chondral lesions, 23 (72%) were bipolar lesions, but only in 12 cases (38%) was bipolar microfracturing necessary. The bipolar lesions treated were located at the centre of the humeral head and glenoid, and their sizes were big enough to locate at least 2–3 microfracture spots. Since the sizes of the lesions were only documented in half of the cases and in varying descriptions, they were not taken into consideration. Accompanying this treatment, 13 patients (41%) received a subacromial decompression, 5 (16%) a resection of the AC joint, 6 (19%) a tenotomy of the long head of the biceps tendon, 1 (3%) a tenodesis of the long head of biceps tendon and 2 patients (6%) received a SLAP (superior labral tear anterior to posterior) repair. These treatments were performed according to preoperative clinical symptoms.

### Surgical techniques and rehabilitation

**Fig. 2:**
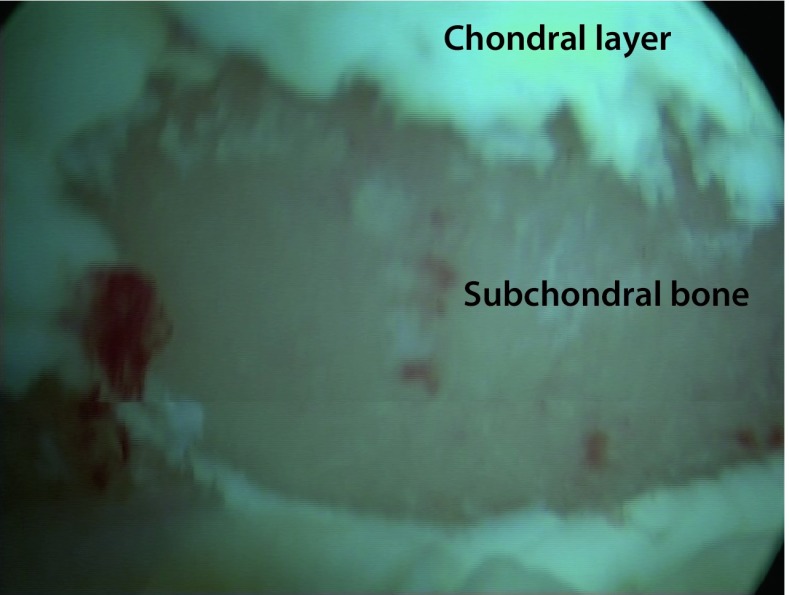
Intraoperative findings of a chondral lesion stage IV according to Outerbridge. Patient in beach chair position, view from the anterior portal onto the humeral head

The senior author performed all of the operations described here. Microfracture was performed in standard arthroscopy according to Steadman [17]. Firstly, the lesions were debrided (see Fig. 2), and as many perforations as possible were then set with a curved awl, at a distance of 3–4 mm apart, until light bleeding occurred. All patients received a standardized physiotherapy program with early functional physiotherapy in a pain-adapted range of motion. Continuous Passive Motion (CPM) was not used.

### Radiological evaluation

Patients received x‑ray preoperatively and at the time of follow-up (anteroposterior, outlet and axillary views). For the retrospective analysis, preoperative radiographs were available from 31 out of 32 patients (one series was not available). The evaluation of osteoarthritis grade was classified according to the Samilson’s classification [13].

MRI (open, 0.25 T) was performed preoperatively and during the follow-up evaluation in order to quantify the quality of the chondral layer in the joint (data not shown). Unfortunately, the MRI results showed a large variation and variety of possible interpretations. Subsequently, the chondral layer was not sufficiently evaluable, and those images were not included in the analyses.

### Statistics

SPSS 19.0 (IBM Company, Armonk, NY, USA) was used for statistical analysis. The Wilcoxon signed-rank test was used for comparison within groups and the Mann–Whitney U test for comparison between groups. The level of significance was marked at *p* ≤ 0.05.

## Results

The mean follow-up time was 105 months (64–147 months). The mean time until shoulder arthroplasty was required in these 5 patients was 47 months (5–79 months; three hemi and two total-shoulder arthroplasties). The results of the descriptive analysis of the 5 patients did not show any significant differences to the main group (mean age 57 versus 56 years, *p* = 0.896).

### Clinical results

No pain at all in the operated shoulder was reported by 13 patients (41%) at the time of follow-up; 12 (38%) patients experienced moderate pain at a higher activity level and 7 (22%) during normal motions of the shoulder joint. Pain at night was still experienced by 6 (19%) patients and 3 (9%) experienced pain at rest. Furthermore, the patients who experienced pain stated that this was only from time to time, and always to a moderate and bearable degree. At the time of follow-up, 19 patients (59%) were satisfied or very satisfied with the outcome of the intervention.

The mean ranges of motion of the affected shoulders are documented in Table 1. There was no statistically significant difference in the ability to elevate, abduct or rotate the shoulder externally in the comparison between the preoperative situation and the time of follow-up. Only the internal rotation increased to a statistically significant degree from the height of the iliosacral joint to L1 (*p* = 0.033). Regarding the CMS and DASH Score, there was no statistically significant difference between the operated and the healthy shoulder at the time of follow-up. Detailed findings are reported in Table 2. There were no statistically significant differences between the shoulders for: overall CMS (*p* = 0.462), relative CMS (*p* = 0.896), SSV (*p* = 0.586), pain level (*p* = 0.721) and DASH Score (*p* = 0.097).

**Table 1:** Results in the range of motion of the operated shoulder pre- and postoperatively

	Elevation	Abduction	IRO	ERO
Preoperative	140° ± 53°	144° ± 57°	ISJ ± 32°	45° ± 22°
Postoperative	153° ± 49°	168° ± 8°	L1 ± 11°	49° ± 21°

*IRO* internal rotation, *ERO* external rotation, *ISJ* iliosacral joint, *L1* lumbal spine L1

**Table 2:** Results in Constant–Murley Score (CMS), subjective shoulder value (SSV) and Disabilities of the Arm, Shoulder and Hand (DASH) Score of the operated and non-operated shoulder at the time of follow-up examination

	Operated shoulder	Non-operated shoulder
CMS	74 ± 26 points	75 ± 28 points
Relative CMS	95 ± 14%	95 ± 31%
Pain value	12.9 ± 3 points	13 ± 3.7 points
SSV	86 ± 13%	88 ± 19%
DASH	12 points	8 points

**Fig. 3:**
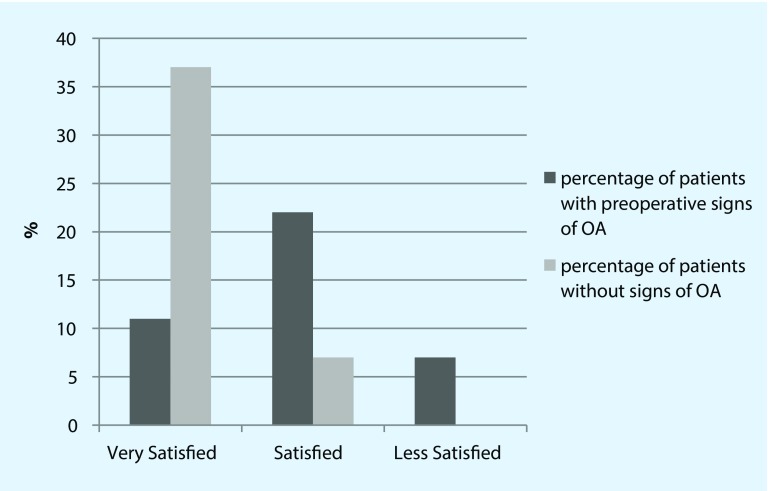
Satisfaction of patients with and without any preoperative signs of osteoarthrosis (*OA*)

In order to evaluate the influence of preoperative signs of osteoarthritis before the treatment, patients were divided into two groups with or without preoperative radiological signs of osteoarthritis. There was no significant difference concerning the distribution of age, trauma in the anamnesis and overall CMS. Significant differences were observed in relative CMS, DASH Score, pain during activity and satisfaction (see Table 3 and Fig. 3).

**Table 3:** Comparison between the groups with or without preoperative osteoarthritis (OA)

	Preop. OA	Preop. no signs	Significance
Mean age (years)	56	55	*P* = 0.976
Trauma preop (*n*)	4	2	*P* = 0.449
CMS	76 ± 14 points	72 ± 35 points	*P* = 0.413
Relative CMS	89 ± 15%	102 ± 8%	*P* = 0.019*
DASH	15 points	9 points	*P* = 0.037*
Pain during activity	8/11	1/11	*P* = 0.007*
Satisfaction	3 very/6 satisfied/3 less	10 very/2 satisfied	*P* = 0.016*

*CMS* Constant–Murley Score, *SSV* subjective shoulder value, *DASH* Disabilities of the Arm, Shoulder and Hand. Preop preoperative

* = *P* < 0.05

Furthermore, data were analysed according to the question of whether patients with radiologic progression of osteoarthritis had a different outcome to those without progression in the x‑ray (mean age of 57 vs. 54 years, *p* = 0.494). In CMS and DASH Score, there were no statistically significant differences between the groups (overall CMS 83 ± 12 vs. 63 ± 35 points, *p* = 0.121; relative CMS 98 ± 13% vs. 91 ± 14%, *p* = 0.242; DASH Score 13 ± 16 vs. 13 ± 11 points, *p* = 0.552). A previous shoulder injury also had no statistically significant influence on the outcome. The distribution of age was again similar in those groups (mean age of 53 years with a previous injury, 57 years without, *p* = 0.233). The overall CMS in the group with a previous injury was 78 ± 19 points, the relative CMS 89 ± 17% and DASH Score 7 ± 3, versus an overall CMS of 73 ± 29 points (*p* = 0.674), a relative CMS of 97 ± 12% (*p* = 0.223) and a DASH Score of 10 ± 14 points (*p* = 0.392) in the group without a previous injury.

Patients with bipolar lesions showed statistically significantly lower results in the Subjective Shoulder Value (SSV; mean SSV = 80.6 in the group with bipolar lesions and SSV = 94.7 in patients with unipolar lesions, *p* = 0.039). There were 19 patients with bipolar lesions (mean age 54 ± 9 years) and 8 patients with unipolar lesions (mean age 61 ± 8 years; seven glenoidal and one humeral unipolar lesion).

### Radiological results

The results of the x‑ray evaluation are shown in Table 4. Progression of osteoarthritis was suffered by 13 patients (57%), which was found to be statistically significant (*p* = 0.002).

**Table 4:** Radiological results of all patients

	X-ray Samilson 0	X-ray Samilson I	X-ray Samilson II	X-ray Samilson III	Glenohumeral distance	Acromiohumeral distance
Preop	*N* = 14	*N* = 10	*N* = 5	*N* = 2	2.6 ± 2.3 mm	6.5 ± 5.2 mm
Follow-up	*N* = 5	*N* = 8	*N* = 8	*N* = 2	2.4 ± 1.9 mm	7.4 ± 2.9 mm
Follow-up patients preop Samilson 0	*N* = 1	*N* = 2	*N* = 6	*N* = 2	–	–

In the separated groups, with and without preoperative signs of osteoarthritis in the x‑rays, patients with preoperative signs of osteoarthritis had a progression to higher stages according to Samilson than those without preoperative signs (*p* = 0.013). Those patients without preoperative signs of osteoarthritis in initial x‑rays were significantly more content with the result (*p* = 0.003) and reported less pain during activity (*p* = 0.031).

## Discussion

After a mean follow-up time of 105 months, 13/27 patients reported no pain, 12/27 patients moderate pain. Of these 12, 6/27 reported pain only at night and 3/27 only during rest. Regarding the outcome of surgery, 19/27 patients indicated that they were “satisfied” or “very satisfied”. There were no differences in the ranges of motion pre- and postoperatively, except for a statistically significant increase in internal rotation. Patients without any signs of osteoarthritis before the operation showed statistically significantly better outcomes. There was a statistically significant increase in radiological signs of osteoarthrosis in pre- versus postoperative patients. Patients with bipolar lesions showed statistically significantly poorer SSV results.

Microfracturing has been described as a treatment option for chondral defects in various studies. The procedure for microfracture in the knee was first published in 1999 [3, 16–18], with promising long-term results observed in the knee and talus [6, 10, 12]. However, to the best of our knowledge, only a few studies deal with this procedure in the shoulder, with a notable lack of studies investigating the long-term effects of microfracture in this joint. There have been studies with promising results but very low numbers of patients. A follow-up on 5 patients treated with a combination of microfracture and periostal flap (because of a chondral defect of the humeral head) revealed a good clinical outcome and pain reduction after 26 months [14]. Another cohort of 8 patients treated with microfracture of the shoulder showed a significant increase in CMS after 15 months, as did another group of 16 patients retrospectively reviewed after 28 months [2, 15].

Comparing our results to these previous studies, this study, to the best of our knowledge, describes the longest duration of follow-up on the largest cohort of patients that has been published to date. Here, 5 patients underwent total or partial shoulder arthroplasty after a mean time of 47 months after microfracture, which was still a subjective benefit for younger patients. According to the results of earlier studies, we also noted that even though the clinical outcome after microfracture was good, the radiographs showed a progression of osteoarthritis. Nevertheless, as shown in this study, many patients profit from the procedure with regards to pain and clinical outcome. Factors like pain and shoulder dysfunction are the main indications for further treatments such as total or partial shoulder arthroplasty. Therefore, the main goal of any treatment option prior to these interventions should focus on maintaining a bearable status of the shoulder joint for as long as possible. A treatment like microfracturing seems to be a good option to delay the onset of symptoms and maintain shoulder function.

For prediction of the outcome of microfracturing, it should be taken into consideration that bipolar lesions seem to result in worse postoperative results. A study performed on 31 shoulders revealed the best postoperative outcome in patients with isolated lesions compared to a group with bipolar lesions after 47 months [8]. In our study, patients with unipolar lesions achieved statistically significantly better SSV results compared to patients also having bipolar lesions. No statistically significant differences in the groups with bipolar and unipolar lesions were seen for the range of motion and subjective pain and functionality questions. Nevertheless, unipolar lesions seem to represent the best indication for the treatment and patients with bipolar lesions should be informed of the higher postoperative risk of ongoing symptoms.

It should be noted that in this study, minor side pathologies other than the microfracture were treated during the arthroscopy. This has been reported in many of the previous studies. There is no doubt that treatment of side pathologies concurrently with the microfracture can have a major impact on the outcome after these surgeries. However, there are few cases with clearly isolated chondral lesions without any other pathology in the glenohumeral joint. Leaving an obvious pathology in the joint untreated influences the outcome after microfracture to at least the same degree. Furthermore, it is not in the patient’s best interest to leave a pathology untreated with the knowledge that this could lead to further pain and dysfunction. Ultimately we have to accept that microfracture is only one part of a complex treatment of the degenerated glenohumeral joint.

## Practical conclusion

Microfracturing of the glenohumeral joint does not stop the natural process and progression of osteoarthrosis, but the clinical outcome is still satisfying for the patients. The patients studied here reported only few symptoms, most notably less pain for 5 years or more. The radiological findings show that microfracturing does not prevent radiographic progression of osteoarthritis. In cases of early chondral lesion without any radiological signs of osteoarthritis, the clinical outcome was best. Patients with bipolar lesions have lower SSV scores. According to the results presented, arthroscopic treatment with microfracture should be taken into consideration as part of a complex treatment of the degenerated glenohumeral joint.
